# Potential Biological Markers of Atrial Fibrillation: A Chance to Prevent Cryptogenic Stroke

**DOI:** 10.1155/2017/8153024

**Published:** 2017-07-13

**Authors:** István Szegedi, László Szapáry, Péter Csécsei, Zoltán Csanádi, László Csiba

**Affiliations:** ^1^Department of Neurology, Clinical Centre, University of Debrecen, Debrecen, Hungary; ^2^Department of Neurology, Clinical Centre, University of Pécs, Pécs, Hungary; ^3^Department of Cardiology, Clinical Centre, University of Debrecen, Debrecen, Hungary

## Abstract

Stroke affects millions of people all over the world, causing death and disability. The most frequent type of this disease is ischemic stroke, which can be caused by different factors. In approximately 25 percent of cases, no obvious cause can be found. Recent observations have shown that paroxysmal atrial fibrillation could be responsible for a significant number of cryptogenic stroke events. Short- or long-lasting ECG monitoring could help with the diagnosis of transient arrhythmias. Unfortunately, these techniques either are expensive or require good patient compliance. An alternative option is the identification of biological markers that are specific for atrial fibrillation and can be used to predict arrhythmia. In this review, we give a summary of the recent advances in the research of arrhythmia markers. Based on their structure and function, we differentiated four groups of biomarkers: markers of inflammation, markers of fibrosis, markers with hormonal activity, and other markers. In spite of intensive researches, the optimal biological marker is still not available, but there are some promising markers, like NT-proBNP/BNP.

## 1. Introduction

Stroke is a common vascular disease manifesting in different subtypes, causing death and disability, and therefore it is an important challenge for healthcare systems. After an ischemic event, a thorough workup identifies the cause in about 75 percent of cases. In the remaining 20–25 percent, no causative factor can be found: these are called cryptogenic stroke events. In about 25–30 percent of these events, the underlying cause could be paroxysmal atrial fibrillation (PAF) [1–3]. Most studies defined PAF as an event that lasts longer than 30 seconds, referring to the AHA 2006 guidelines [4]. In atrial fibrillation (AF), the blood flow in the cavities of the heart is turbulent, which can precipitate thrombus formation and embolization [5]. Despite the fact that paroxysmal atrial fibrillation is frequently asymptomatic, the risk of stroke is the same as in permanent AF [6]. The diagnosis requires extended ECG monitoring (e.g., Holter ECG, outpatient monitoring [7, 8]) of the patient. If a cardiogenic mechanism is identified behind a stroke event, anticoagulation is warranted instead of antiplatelet therapy [4]. Poststroke patients, however, are not optimal candidates for long-lasting ECG monitoring (insufficient compliance, frequent falls, aphasia, limb paresis, etc.). There is an increasing need for blood biomarkers that are capable of identifying patients at a significant risk of PAF. Currently, the optimal marker is still unknown. An ideal biological marker has the following characteristics: high sensitivity, high specificity, high predictivity, and rapid, simple, accurate, inexpensive, and reproducible detection in all relevant patients. In this review article, we aimed to summarize the most important recent observations.

## 2. Methods

PubMed, EMBASE, BioMed Research International, and Google Scholar were searched for published studies. We examined studies that have presented data on the association between atrial fibrillation and biological markers. We used the keywords “atrial fibrillation”, “biological marker”, and “stroke” and the names of the markers. Based on their structure and function, four groups of biomarkers can be differentiated: markers of inflammation, markers of fibrosis, markers with hormonal activity, and other markers.

## 3. Markers of Inflammation

In the last few years, a number of trials have concluded that AF is triggered by inflammation, resulting in electrical and structural remodeling [9, 10]. Active inflammation can provoke AF, which in return causes an inflammatory response that further enhances atrial remodeling, resulting in arrhythmia, the so-called “AF begets AF” phenomenon. The progress is similar to a spiral: inflammation begets AF, and AF begets inflammation [11].

A new promising inflammatory marker is pentraxin-3 (PTX3), a member of the long pentraxin family. The C-terminal sequence of PTX3 is homologous with serum amyloid P component and the classic short pentraxin CRP. PTX3 is produced in large amounts by different cells (e.g., monocytes, macrophages, and endothelial cells) in local inflammatory lesions, whereas CRP is mostly produced by the liver [12].

A subgroup of 382 patients, who were selected from 36 centers with AF in their history but in sinus rhythm at randomization, was enrolled in the GISSI-AF biohumoral study that investigated the association between the plasma concentrations of pentraxin-3 (PTX3), high-sensitivity C-reactive protein (hsCRP), and interleukin-6 (IL-6), the echocardiographic findings, and the time of the first return of AF [13]. Recurrent AF developed in 204 patients during the one-year-long follow-up. Baseline plasma concentrations of IL-6, hsCRP, and PTX3 were measured and no significant differences between patients with or without recurrent AF were found. At 6 and 12 months of follow-up, IL-6 and PTX3 concentrations were significantly higher in patients with AF compared to those who were in sinus rhythm, and the hsCRP levels were higher in patients with the most recent episodes of AF. Baseline levels of PTX3, IL-6, and hsCRP were not significantly associated with a higher risk of AF recurrence. These markers can be elevated in AF patients, but they were found to be weak predictors of arrhythmia recurrence in this study.

The neutrophil-to-lymphocyte ratio (NLR) is a major marker of subclinical inflammation, and it is a widely investigated marker recently used in the prediction of cerebrovascular diseases [14, 15].

Ertaş et al. carried out a retrospective study on 126 consecutive nonvalvular AF patients with or without thromboembolic stroke [16]. A group of 24 patients in sinus rhythm served as a reference point for the comparison with the AF group. Based on NLR values at admission, the study population was divided into tertiles. A low NLR level (*n* = 84) was defined as a value in the lower two tertiles and a high NLR level (*n* = 42) was defined as a value in the third tertile. Stroke rates and CHADS2 scores were found to be significantly higher in the high NLR group compared to the low NLR group. Stroke patients had significantly higher mean white blood cell (WBC) counts and NLR values. Therefore, NLR can be used in patients with nonvalvular AF as an independent predictor of thromboembolic stroke.

A cohort study conducted by Saliba et al. aimed to find the association between NLR, atrial fibrillation, and stroke [17]. 32,912 adult patients with AF, with no anticoagulation therapy at the baseline, and without previous stroke or TIA were analyzed retrospectively in a computerized database. The patients were followed up for the first occurrence of stroke or TIA from 1 January 2012 until 31 December 2012. 981 subjects developed stroke during a follow-up of 30,961 person-years (stroke rate: 3.17 per 100 person-years). The patients were grouped into quartiles based on NLR levels. The incidence rate of stroke increased in a dose-response manner across NLR quartiles, and therefore the study showed that there is a significant association between NLR level and the first episode of stroke in patients with atrial fibrillation.

Based on these studies, NLR is a promising marker in predicting paroxysmal AF, while the usefulness of PTX3 is questionable (Table 1).

## 4. Markers of Fibrosis

Galectin-3 (Gal-3) is a *β*-galactoside-binding lectin that seems to play a major role in the regulation of fibrosis and inflammation [18].

Ho et al. investigated the relation between plasma galectin-3 concentrations and the incidence of AF. Plasma levels of the peptide were measured in 3,306 members of the Framingham Offspring cohort (who participated in the sixth examination cycle between 1995 and 1998) [19]. They used Cox proportional hazard regression models to evaluate the association between baseline Gal-3 concentrations and the incidence of AF. They found that elevated Gal-3 plasma levels were associated with a higher risk of developing AF, but after adjusting for clinical risk factors to predict AF risk, the association was no longer significant [20].

An observational study managed by Gurses et al. also aimed to find the correlation between plasma galectin-3 levels and atrial fibrillation [21]. Seventy-six patients with paroxysmal or persistent AF and preserved left ventricular systolic function and 75 age- and gender-matched control patients were enrolled in the study. All of them underwent transthoracic echocardiographic examination (TTE) to measure left atrium (LA) size and left ventricular (LV) function to exclude any structural disease of the heart. LA volume index (LAVI) was calculated on the basis of the patient's body surface area. Galectin-3 level was measured and it was significantly elevated in patients with AF in comparison with the control group. Serum galectin-3 levels were also significantly higher in patients with persistent AF than in those with paroxysmal AF. Multivariate regression analysis was performed demonstrating that serum galectin-3 levels and LAVI were independent predictors of AF. Gurses et al. found an independent correlation between LAVI and serum galectin-3 levels in patients with AF through linear regression analysis.

The observational study of Yalcin et al. was very similar, but this investigation was conducted using delayed enhancement magnetic resonance imaging (DE-MRI) to estimate the degree of atrial fibrosis and atrial electromechanical delay (AEMD), a noninvasive echocardiographic method to measure inter- and intra-atrial conduction delays [22]. Thirty-three patients with paroxysmal AF and unimpaired LV function were enrolled. The study demonstrated that serum galectin-3 levels had an independent correlation with the extension of LA fibrosis demonstrated by DE-MRI in paroxysmal AF patients. Serum galectin-3 levels also had a correlation with intra-left- and inter-AEMD, which is really important because they are accepted as noninvasive echocardiographic markers of atrial electrical remodeling.

TGF-b1 is expressed in endothelial cells, vascular smooth muscle cells, and myofibroblasts [23]. In the heart, TGF-b1 seems to be a factor that causes different diseases by inducing cardiac fibrosis, based on studies examining overexpression and knockout models [24]. In a mice study, the animals with increased expression of TGF-b1 were prone to develop atrial fibrillation as a result of a higher degree of atrial fibrosis [25].

TGF-b1 levels seem to be increased in humans with atrial fibrillation as well. In a study, Lin et al. examined the relation between TGF-*β*1 and atrial fibrillation in patients with essential hypertension (EH) [26]. 75 patients with AF secondary to EH were selected in the study and then divided into 2 subgroups: the paroxysmal AF group (pAF) consisting of 44 patients and the chronic AF group (cAF) consisting of 31 patients. 37 EH patients with sinus rhythm (SR) were selected in the EH + SR control group and 36 healthy subjects were assorted as normal controls (NC group). Clinical characteristics of the patients were collected and TTE examinations were also performed. Blood samples were taken in the morning from fasting and resting subjects for the assessment of TGF-*β*1 and CTGF (connective tissue growth factor) levels. TGF-*β*1 and CTGF serum levels were significantly higher in the EH groups than in the NC group. TGF-*β*1 and CTGF levels were the highest in the cAF group, followed by the pAF and SR groups. Lin et al. did not find significant differences in TGF-*β*1 and CTGF levels between the pAF group and the cAF group. In AF patients, there was an independent correlation between serum levels of TGF-*β*1 and left atrial diameter (LAD), the presence of AF, aldosterone, CTGF, and age. As a possible conclusion, serum TGF-*β*1 was found to indicate the synthesis of CTGF causing enlargement and remodeling of the left atrium, which can lead to AF in EH patients.

Matrix metalloproteinase-9 (a member of the matrix metalloproteinase family) is an endopeptidase, synthesized and secreted in monomeric form as zymogen. It can degrade components of the extracellular matrix and it also takes part in various physiological and pathological processes including development, growth, and reproduction and, additionally, vascular, proliferative, and inflammatory diseases [27].

In their study, not only did Li et al. measure the plasma level of MMP-9, but also they investigated its significance in different stages of idiopathic AF [28]. The patients were categorized into 3 groups: paroxysmal AF, persistent AF, and permanent AF groups, each containing 25 patients. The control group consisted of 40 healthy individuals. Venous blood samples were taken. MMP-9 plasma levels in the AF groups showed significant elevation compared to the control group. From paroxysmal AF through persistent AF to permanent AF, the plasma levels of MMP-9 showed a significant gradual increase.

The growth/differentiation factor 15 (GDF-15) protein is a member of the transforming growth factor beta superfamily. It has several physiological functions including the regulation of proliferation and apoptosis in normal, injured, and transformed cells, but it also has pathological functions such as growth inhibition and overexpression in cancer cells [29]. Shao et al. aimed to find the correlation between the serum levels of GDF-15, NRG-1, and nonvalvular AF. Their study included 67 patients with nonvalvular AF and 67 healthy persons matched for age, sex, and atherosclerotic risk factors [30]. They collected baseline demographic and clinical characteristics and performed TTE. They measured the plasma levels of GDF-15, NRG-1 (a member of the epidermal growth factor (EGF) gene family playing a role in growth, cell survival, cardiovascular development, and metabolism [31]), and other basic laboratory parameters. Patients from the AF group had higher GDF-15 and NRG-1 levels and LAD values than non-AF patients. Patients with paroxysmal AF had a significantly higher serum level of GDF-15 compared to the control group. Likewise, NRG-1 levels were also higher in paroxysmal AF patients. According to multivariable analyses, GDF-15 was independently associated with paroxysmal AF.

Sonmez et al. examined a study population consisting of 52 patients diagnosed with nonvalvular AF and 33 age-matched subjects without AF in their history [32]. Their goal was to compare the serum levels of novel biomarkers between a group of AF patients and a group of healthy individuals. These markers were galectin-3, MMP-9, lipocalin-2 (NGAL, a novel adipokine associated with insulin resistance [33]), PIIINP (the amino-terminal peptide of type III procollagen, released into the blood during both synthesis and degradation of collagen type III [34]), hsCRP, and NLR. Correlation analyses found a significant correlation between NLR and LAVI (left atrial volume index), but not between hsCRP and LAVI. There was a strong correlation between galectin-3, MMP-9, PIIINP, and LAVI. MMP-9, galectin-3, and PIIINP levels were significantly higher in AF patients, but NGAL levels were not. NLR and hsCRP levels were also elevated in AF patients.

As a part of the Cardiovascular Health Study, Rosenberg et al. assessed the plasma levels of 2 fibrosis biomarkers, PIIINP and TGF-*β*1 [35]. PIIINP levels were measured in 2,935 participants, of whom 767 developed AF. PIIINP levels showed a nonlinear relationship with the risk of incident AF both before and after risk adjustment. A linear relationship was observed between the risk of AF and PIIINP levels approximately up to the median value, but unfortunately no significant association was identified beyond that point. TGF-*β*1 levels were assessed in 1,538 individuals with 408 cases of incident AF, but this marker's levels were not associated with AF risk. No association was found between TGF-*β*1 levels and the incidence of AF in unadjusted or adjusted models.

To summarize, galectin-3, MMP-9, GDF-15, and PIIINP are promising markers in the prediction of paroxysmal AF, while TGF-*β*1 shows a lower potential (Table 2).

## 5. Markers with Hormonal Activity

Natriuretic peptides (NPs) are produced in the heart and released into the circulation in response to pressure and volume overload. NP levels provide information about the systolic and diastolic function as well as the right ventricular and valvular function [36]. In recent years, different NPs, such as brain natriuretic peptide (BNP), its N-terminal prohormone (NT-proBNP), and atrial natriuretic peptide (ANP), emerged as possible biological markers of atrial fibrillation. The connection between BNP and/or NT-proBNP has been investigated by numerous studies. Silvet et al. measured BNP levels in 72 outpatients with chronic atrial fibrillation (AF) and in 49 control subjects without AF. BNP levels were significantly higher in patients with AF [37]. Another study was performed at the Kagawa University School of Medicine Hospital that aimed to measure BNP levels in patients with acute ischemic stroke [38]. This cohort included 99 patients with acute cerebral infarction. 23 patients were excluded due to having myocardial infarction, heart failure, valve disease, or chronic renal failure. 36 of the remaining patients developed cardioembolic stroke with atrial fibrillation (23 with permanent and 13 with paroxysmal atrial fibrillation) and 40 had noncardioembolic stroke. BNP was evaluated on the first morning after admission, and TTE was also performed. In the cardioembolic stroke/atrial fibrillation group, plasma BNP levels, LAD, and the ratio of peak early filling velocity to peak atrial systolic velocity (E/A) were significantly increased, while left atrial appendage flow was significantly reduced compared to noncardioembolic stroke patients. First-day BNP and LAA flow were useful in differentiating cardioembolic stroke with AF from noncardioembolic stroke.

In the Cardiovascular Health Study (CHS), Patton et al. found a connection between AF and NT-proBNP [39]. NT-proBNP levels were measured in 5,447 patients (2 of them were excluded due to missing baseline ECG results). NT-proBNP levels showed a strong association with prevalent AF. After a median follow-up of 10 years (maximum of 16 years), 1,126 cases of incident AF were registered (a rate of 2.2 per 100 person-years). NT-proBNP levels proved to be greatly predictive of incident AF.

Within the settings of the Multi-Ethnic Study of Atherosclerosis (MESA), 5,518 patients were enrolled to investigate a possible association between serum NT-proBNP levels and AF [40]. NT-proBNP levels were measured from frozen serum samples drawn at enrollment. The associations between NT-proBNP and gender, age, and ethnicity/race were also investigated. Patton et al. followed up the patients for a median of 7.6 years. During this time, 267 of them developed AF. The average NT-proBNP level was higher in subjects with AF. NT-proBNP was statistically significantly associated with incident AF.

Kara et al. investigated the association of BNP with incident AF in a large population-based cohort study [41]. The patients did not have a history of prior stroke, coronary heart disease, heart-device therapy, open heart surgery, or prevalent AF at baseline. 3,067 subjects were involved in the logistic regression analysis. They examined the association of BNP as a continuous and binary variable (with predefined gender-specific BNP thresholds) with new onset of AF as well as its value for risk prediction beyond traditional AF risk factors. Higher BNP levels showed association with excessive incidence of AF (e.g., resulting in a fourfold risk in a five-year period for subjects with a BNP level over 31 pg/mL for men and 45 pg/mL for women in the crude model). After adjustment for traditional AF risk factors and CAC (coronary artery calcium), the association stayed statistically significant. The associations were more explicit in younger patients. To summarize these results, elevated levels of BNP were found to be associated with significant excess of incident AF independently of traditional risk factors of AF in the general population. In the future, the gender-specific BNP thresholds may help in the detection of subjects with unknown or future AF that can lead to stroke events.

Rodríguez-Yáñez et al. performed an interesting prospective study enrolling patients with cryptogenic stroke from February 2008 to July 2011 according to the TOAST criteria [42]. Of the 1,050 evaluated patients, 264 were qualified for the study. Blood samples were taken within the first 24 hours from the onset of the stroke for the measurement of proBNP levels. Then, the patients were followed up at 3 and 6 months by a neurologist and later by a primary care physician for 2 years in order to register the development of AF. Fifteen patients (5.6%) developed AF during the follow-up period. Patients who developed AF were older and more frequently had a history of hypertension. Forty-eight patients (18.2%) died during the follow-up period. Higher NT-proBNP levels were detected in patients who developed AF, compared with those who did not. Based on these findings, high NT-proBNP levels measured during the acute phase of stroke in cryptogenic stroke patients are associated with a fivefold increase in the risk of developing AF in the following 2 years.

The TARGET AF was a prospective cohort study of stroke survivors performed in the stroke center of Nice University Hospital, which aimed to identify a relevant marker of delayed AF in the selected patients in whom AF was detected by early and prolonged monitoring [43]. 373 patients were included in the study and 53 of them were excluded due to diagnosis of AF at baseline. 20 patients with sinus rhythm at baseline but with AF in their history were also excluded. Plasma BNP was measured in blood samples taken at admission. Holter ECG monitoring was started immediately at admission and was stopped at discharge in all patients. Newly diagnosed AF was documented in 52 patients (AF prevalence of 17.33%) suggesting the association of early and prolonged monitoring with an increased AF detection rate. Plasma BNP values were significantly higher in patients with AF. The use of all examined parameters together did not provide significant additional diagnostic value over BNP (diagnostic properties of BNP level: sensitivity: 98.08%; specificity: 71.37%; negative predictive value: 99.4%). The most important result of the study is that BNP level has a really strong negative predictive value in stroke patients that can be related to delayed AF.

Fibroblast growth factor-23 (FGF-23) is a bone-derived hormone that plays an important role in the homeostasis of phosphate. FGF-23 reduces gastrointestinal phosphate absorption, inhibits the production of 1,25-dihydroxyvitamin D, and promotes urinary phosphate excretion [44, 45].

Mathew et al. examined participants from the Multi-Ethnic Study of Atherosclerosis (MESA) and the Cardiovascular Health Study (CHS) to investigate the relation between FGF-23 and AF. 6,398 patients were from the MESA study, and 1,350 participants were from the CHS [46]. Incident AF was identified using inpatient and outpatient physician claims data, systematic reviews of hospital discharge diagnoses, and study ECGs over 7.7 and 8.0 years of median follow-up. Cox proportional hazard models were used to test associations between FGF-23 and the risk of developing AF. A series of multivariable models were also constructed. In MESA participants and CHS participants, 291 and 229 incident AF events were observed, respectively. In both MESA and CHS participants, higher FGF-23 concentrations were associated with higher unadjusted incidence rates of AF. Later adjustments for demographics and potential confounding characteristics were carried out. In these analyses, each twofold higher FGF-23 concentration was associated with a 41% higher risk of AF in MESA patients and a 29% higher risk of AF in CHS patients, proving that higher circulating FGF-23 concentrations increase the risk of incident AF. Other biomarkers of mineral metabolism, eGFR, urine ACR, and heart failure events were also accounted for, and the associations remained significant. Adjusting for FGF-23 attenuated the association of low eGFR with incident AF in MESA patients, suggesting that FGF-23 may mediate, in part, the known association of CKD with AF.

NT-proBNP (and BNP) was found to be elevated in AF by several studies. Studies that evaluated the connection of the natriuretic peptide with AF in cryptogenic stroke patients were also performed, determining well-defined cut-off values that are able to predict paroxysmal events in cryptogenic patients. FGF-23 is also a promising marker, but currently we do not have enough information about it (Table 3).

## 6. Markers with Other Functions

Circulating procoagulant microparticles (MPs) are small membrane vesicles that are derived from different cells (e.g., platelets, endothelial cells, leukocytes, lymphocytes, and erythrocytes) in response to activation, injury, and/or apoptosis [47]. Platelet microparticles (PMPs) are procoagulant membranous vesicles produced by activated platelets. PMP levels are elevated in stroke, coronary artery disease (CAD), hypertension, and diabetes.

Choudhury et al. aimed to find the correlation between serum platelet microparticle levels and nonvalvular atrial fibrillation [48]. The study had 3 hypotheses: (1) PMP levels are higher in patients with AF compared to levels in both disease control subjects (i.e., patients with diabetes or stroke, CAD, or hypertension who are in sinus rhythm) and healthy control subjects (i.e., patients without cardiovascular diseases who are in sinus rhythm); (2) PMP levels correlate with levels of soluble P-selectin (sP-selectin) which is a platelet activation marker; (3) in patients with AF, PMP levels are related to the underlying factors that contribute to the comprehensive risk of stroke secondary to AF. The study team performed a case-control study of 70 AF patients, 46 disease controls, and 33 healthy control patients. The levels of PMPs and sP-selectin were significantly higher in both AF patients and disease control subjects compared to healthy control subjects, but no difference was found between AF patients and disease control subjects. There was not any difference in PMP levels between patients with paroxysmal and permanent AF and between those who received antiplatelet or anticoagulant therapy (aspirin and warfarin, resp.). A significant correlation was not observed between PMP and sP-selectin levels and the clinical characteristics that contribute to the elevated risk of stroke in patients with AF. Through multiple regression analysis in the combined cohort of AF patients and the disease control subjects, the presence/absence of AF did not prove to be an independent determinant of PMP and sP-selectin levels.

Ederhy et al. also suggested that procoagulant MP levels in the circulation would be increased in AF, so they elaborated a hospital-based case-control study design, involving 45 patients with AF and 90 control subjects: 45 with cardiovascular risk factors and 45 without [49]. The levels of 3 different MPs were screened: platelet-derived MPs, Annexin V-positive MPs, and endothelial-derived MPs. Annexin V-positive MP levels were elevated in patients with AF compared with control subjects with and without cardiovascular risk factors. The levels of platelet-derived MPs and endothelial-derived MPs were similar in patients with AF and control subjects with cardiovascular risk factors but were significantly higher than in control subjects without cardiovascular risk factors. Finally, the presence of AF strongly predicted Annexin V-positive MP levels. Based on these data, circulating procoagulant MPs can be increased in persistent and/or permanent AF and might indicate a hypercoagulable state that could lead to atrial thrombosis and thus thromboembolism.

Asymmetric dimethylarginine (ADMA) is an endogenous inhibitor of nitric oxide synthase (NOS) capable of causing NO deficiency and an increased risk of thrombosis [50]. Cengel et al. investigated patients in three groups: 17 patients whose AF was detected for the first time within the first 24 hours of presentation (group 1), 25 patients with permanent chronic AF lasting at least 1 year or more (group II), and 18 healthy people as the control group (group III) [51]. Plasma ADMA, SDMA, and L-arginine concentrations were compared. In patients with acute onset of AF, ADMA levels were significantly higher when compared to patients with chronic AF and the healthy control group. ADMA levels were higher in all patients with AF than in the control group of healthy people. This information indicates that endothelial dysfunction and a prothrombotic state develop in a very early phase of AF.

MicroRNAs (miRNAs) are a class of 19–25-nucleotide noncoding RNAs with a broad spectrum of functions including the regulation of cellular differentiation, proliferation, development, and death [52]. Different miRNAs with different functions are known, and they are widely investigated, but their value as blood biomarkers is still not clear.

In a study conducted by Liu et al., 105 patients were enrolled for miRNA investigation [53]. 15 participants were selected for in-depth sequencing of plasma miRNAs: 5 people with paroxysmal AF, 5 people with persistent AF, and 5 healthy individuals. The other 90 participants were randomly classified as test subjects using quantitative reverse transcriptase-polymerase chain reaction (qRT-PCR). Blood samples were taken from all enrolled patients to carry out the in-depth analysis. Massively parallel signature sequencing (MPSS) was also performed. 22 specific miRNAs showed dysregulation in each group. Four candidate microRNAs (miRNA-375, miRNA-146a, miRNA-19a, and miRNA-150) were suitable for further investigation in an independent cohort of 90 plasma samples using TaqMan miRNA quantitative reverse transcriptase-polymerase chain reaction (qRT-PCR). The expression levels of these miRNAs were significantly downregulated in patients with AF, but only miRNA-150 demonstrated significant characteristic changes. Its expression levels were reduced by a factor of approximately 17 times in PAF patients relative to controls and a factor of approximately 20 times in PersAF relative to controls. Based on the median expression results of miRNA-150, no significant differences were found between the levels of miRNA-150 among the healthy controls, PAF patients, and PersAF patients. Moreover, no correlation was found between miRNA-150 levels and the presence or absence of antiarrhythmic drug therapy in AF patients. miRNA-150 levels were independently associated with age and LAD. Parallel plasma CRP measurements showed that the levels of CRP were negatively correlated with the plasma levels of miRNA-150.

McManus et al. analyzed the association between circulating miRNAs and AF [54]. 2,445 individuals from the Framingham Heart Study (FHS) were enrolled. The expression of 385 miRNAs isolated from whole blood was measured using TaqMan chemistry-based assays. After the measurement and statistical analysis, the expression of several miRNAs (miR-150-5p, miR-328, miR-331-3p, and miR-28-5p) was found to be negatively associated with prevalent AF. After adjustment for age, sex, isolation batch, RNA quality, concentration, and 260/280 ratio, the association with AF remained significant only with miR-328, which was found in a relatively high number in patients. Unfortunately, after further adjustments for clinical AF risk factors linked to atrial size and/or pathological atrial remodeling, including weight, height, systolic and diastolic blood pressure, antihypertensive medication (including beta-blocker) use, current smoking, prevalent heart failure, myocardial infarction, and diabetes mellitus, this association was attenuated. The association between higher miR-328 and AF is really interesting, because this miRNA promotes atrial electrical remodeling, thus AF by reducing L-type Ca2+ channel density [55].

The potential in using PMPs and ADMA as predictors of AF is promising. MicroRNAs are quite intriguing, because they have a lot of different types, thus a lot of potential, but more investigations are needed in the future (Table 4).

## 7. Conclusion

The investigated markers have different functions; some of them are connected to inflammation (NLR, pentraxin-3, CRP, and IL-6), while others contribute to the fibrosis of the atrium (MMP-9/TIMP, TGF-*β*1, PIIINP, galectin, and CTGF). Inflammation and fibrosis go hand in hand, so the separation of these markers can be difficult sometimes. Some markers have a characteristic hormonal effect (NT-proBNP, FGF-23), and we can find markers that play a role in protein catabolism (ADMA) or posttranscriptional changes (microRNA), but there are some markers with complex functions and structure as well (circulating procoagulant microparticles). Most of the analyzed markers have promising data, but at present none of them fulfills the criteria of an optimal biomarker. NT-proBNP/BNP are the most promising candidates.

## Figures and Tables

**Table 1:** Markers of inflammation.

Biomarker	Trials	Patients	Results	Potential DX efficiency in cryptogenic stroke
Pentraxin-3	1 prospective trial	382	Weak predictor of the recurrence of AF	+−
NLC	2 retrospective trials	126/32912	Useful in predicting stroke in patients with known AF	+

+−: questionable; +: potentially useful.

**Table 2:** Markers of fibrosis.

Biomarker	Trials	Patients	Results	Potential DX efficiency in cryptogenic stroke
Galectin-3	1 prospective trial	3306	↑ Gal-3: ↑ risk of developing AF after adjusting for clinical risk factors: *⦸* significant	+
	1 prospective trial	76	Galectin-3 ↑ in AF compared with the control galectin-3 ↑ in persistent AF compared with paroxysmal AF	
	1 prospective trial	33	Galectin-3 level is an independent correlate of the extent of LA fibrosis in paroxysmal AF patients	

TGF-*β*1	1 prospective trial	75	TGF-b1 ↑ in cAF and pAF group compared with SR group; *⦸* difference in TGF-b1 levels between the cAF group and the pAF group	+−

MMP-9	1 prospective trial	75	MMP-9 levels ↑ gradually from paroxysmal AF through persistent AF, permanent AF	+

GDF-15	1 prospective trial	67	GDF-15 ↑ in paroxysmal AF independently associated with paroxysmal AF	+

Multimarker	1 prospective trial	52	Galectin-3, MMP-9, and PIIINP ↑ in AF	+
	1 prospective trial	2935	PIIINP showed a nonlinear association with incident AF; *⦸* association between circulating TGF-*β*1 levels and incident AF	

+−: questionable; +: potentially useful; ↑ increased; *⦸*: no.

**Table 3:** Markers with hormonal activity.

Biomarker	Trials	Patients	Results	Potential DX efficiency in cryptogenic stroke
NT-pro BNP/BNP	1 prospective trial	72	↑ BNP levels in patients with AF compared to those without AF	++
	1 prospective trial	76	First-day BNP and LAA flow are helpful in differentiating cardioembolic stroke with AF from noncardioembolic stroke	
	1 prospective trial	5445	NT-proBNP was an important predictor of incident AF, also after adjustment for covariates	
	1 prospective trial	5518	NT-proBNP was significantly associated with incident AF and is a strong predictor of it	
	1 prospective trial	3067	↑ BNP levels were associated with significant excess of incident AF and independent of traditional AF risk factors	
	1 prospective trial	264	↑ ProBNP levels determined during the acute phase of stroke ↑ 5-fold the risk of developing AF in cryptogenic stroke patients in the following 2 years	
	1 prospective trial	300	BNP level has a really strong negative predictive value in patients with stroke that can be related to AF	

FGF-23	1 prospective trial	7748	FGF-23 concentrations were associated with higher unadjusted incidence rates of AF ↑ FGF-23 concentration ↑ the risk of incident AF	+

+: potentially useful; ++: very promising; ↑: increased.

**Table 4:** Markers with other functions.

Biomarker	Trials	Patients	Results	Potential DX efficiency in cryptogenic stroke
Circulating procoagulant microparticles	1 prospective trial	70	↑ PMPs in both AF patients and disease control subjects compared to healthy control subjects; *⦸* difference between AF patients and disease control subjects; *⦸* difference in PMP levels between patients with paroxysmal and permanent AF and between those receiving anticoagulant therapy	+
	1 prospective trial	45	Circulating procoagulant MPs can be ↑ in persistent and/or permanent AF and might reflect a hypercoagulable state that could lead to atrial thrombosis and thromboembolism	

ADMA	1 prospective trial	42	ADMA levels in patients with acute AF ↑ compared to patients with chronic AF and healthy controls	+

MicroRNA	1 prospective trial	10	The expression levels of these 4 miRNAs ↓ in patients with AF; the miRNA-150 levels ↓ by a factor of approximately 17 times in paroxysmal AF patients relative to controls and a factor of approximately 20 times in persistent AF relative to controls	+
	1 prospective trial	2445	Circulating levels of miR-328 that were associated with prevalent AF adjustment for risk factors that promote atrial remodeling attenuated the association	

+: potentially useful; ↑: increased; ↓: decreased; *⦸*: no.
